# Green Composites Based on Animal Fiber and Their Applications for a Sustainable Future

**DOI:** 10.3390/polym15030601

**Published:** 2023-01-24

**Authors:** Guravtar Singh Mann, Naved Azum, Anish Khan, Malik Abdul Rub, Md Imtaiyaz Hassan, Kisa Fatima, Abdullah M. Asiri

**Affiliations:** 1 Department of Mechanical Engineering, Lovely Professional University, Phagwara 144402, India; 2 Center of Excellence for Advanced Materials Research, King Abdulaziz University, Jeddah 21589, Saudi Arabia; 3 Chemistry Department, Faculty of Science, King Abdulaziz University, Jeddah 21589, Saudi Arabia; 4 Centre for Interdisciplinary Research in Basic Sciences, Jamia Millia Islamia, Jamia Nagar, New Delhi 110025, India; 5 Department of Biotechnology, Jamia Millia Islamia, Jamia Nagar, New Delhi 110025, India

**Keywords:** green composites, chicken fibers, silk, human hair, wool

## Abstract

Global climate change is already affecting the environment, as glaciers are receding, ice on rivers and lakes is melting, plant and animal range`s have altering, and trees are blooming early. Therefore, focus has shifted towards sustainable materials. There is a growing desire for materials that have a unique combination of qualities that metals, polymers, and other materials cannot provide, therefore scientists are turning their focus to green composites. Green composites offer a wide range of uses in automotive, aerospace, and marine applications. Composites are multiphase resources with separate interfaces that contain chemically different materials. Composites are made up of a variety of materials that are distinct in nature, and they give a set of desirable features that are superior to those of their predecessors or parents. Natural fibers are less expensive, more readily available, rust-resistant, plentiful, nontoxic, and safe for human skin, eyes, and respiratory systems. Green composites are created by combining renewable fibers with polymers (matrix) to create a new class of composites known as “green composites.” This review includes studies on various animal-based fibers and their applications. In this article, recent advancements in the field of these fibers and their composites of fibers are also discussed. The physical, chemical, and mechanical properties are also discussed in this paper. Moreover, the benefits and drawbacks of using these fibers are also discussed in detail. Finally, the paper gives an outline of the topic. The results from composites constructed from each fiber are provided, along with appropriate references for more in-depth analysis studies. This review is specially performed to strengthen the knowledge bank of the young researchers working in the field of natural composites.

## 1. Introduction

The green composites derived from renewable resources have the potential to provide benefits to corporations and the natural environment due to dwindling petroleum resources. Moreover, natural fibers are known as eco-sustainable fabrics with many positive properties as compared to synthetic fibers. Cellulosic and synthetic fibers have largely dominated the fabrics produced in the industry, but animal protein fibers still have their importance when the category of textile demands high-quality fabric in the fashion market. Some examples of animal fibers include wool, cashmere, mohair and camel, llama, alpaca, vicuna, and rabbit hair, as these are made up of complex proteins [1]. The structural fibrous proteins make up the components of animal fibers. They can be found in various parts of an animal’s body, including fur, tissue, cartilage, skin, arteries, and muscles in mammals, and cuticles and silks in arthropods. The protein of these fibers is built up by specific amino acids [2]. These animal fibers are biodegradable as well as having low density, low cost, and are readily available with a continuous supply and safe for handling [3]. The customers’ present demand is to wear comfortable, smooth, soft products with fashionable aesthetic. Therefore, animal fibers play a significant role in coping with the demand of such customers. The fibers obtained from various animals like goats, camels, and yak are now leading the fabric industry. Therefore, cashmere, camel, and yak hair demand has risen to two to three times that of the raw material supply [4,5]. Table 1 highlights the different properties of natural fibers derived from plants and animals.

Camel hair has a high demand due to its luxurious texture, therefore the cost is quite high due to the scarcity [16,17,18]. The demand for the product at a higher rate requires the researcher to carry out the research and handle the fiber in an effective way to achieve a superior quality of the textile material. These fibers are mostly used in textile products. Some of the unique characteristics of the camel hair show its ability to modify and improve the quality and appearance of the product. Some of the characteristics include good strength, warmth, natural colors, and luster. One essential mechanical property, tensile strength, may determine the efficiency and quality of manufactured fine hair fibers [19]. Xiao et al. [20] studied the tensile behavior of the camel hair fiber under dry, wet, halide and ionic treatment. It was observed that the composite model of the fiber contained discrete crystalline phases combined with the amorphous fibrils’ matrix and mechanical properties of the hair affected by fibrils and the matrix [21,22]. Fibrils control the crystalline phase of the hair structure. The tensile relaxation behavior of the hair fiber can be analyzed by the composite organizations, which consist of amorphous globules and microfibrils enriched with disulfide bonds at the elastic stretching region. Xiao et al. [23] examined an experimental and analytical tensile relaxation model of camel hair to interpret the tensile strength of camel hair under dry as well as wet conditions. The analytical framework was designed using a lithium bromide or sodium bisulfite solution to modify the spring portion of an elastic spring and a viscous dashpot [24]. Silk fiber is a protein-rich fiber with a distinctly crystalline nature and biodegradable properties. It can withstand high strength and stiffness under tension and compression. It is one of the strongest natural fibers. Its application is focused on the fields of manufacturing, biotechnology, and cosmetics [25].

In comparison to tussah silk fiber, B. mori silk fiber can withstand a high loading of 10N, whereas tussah can withstand only up to 2N, but has greater elongation (i.e., 89%) properties than B. mori silk fiber. With a Young’s modulus of 6 GPa, tussah fiber can withstand a higher rate of ultimate tensile strength of about 250 MPa. This may be attributed to molecular shifts and the well-aligned interconnection between the crystalline and non-crystalline regions. The chances of fiber failure are higher in the disordered field [26,27]. B. mori silk consists of a high proportion of crystalline structure, whereas a high proportion of amorphous structures is observed for the tussah silk fiber. Yak fiber epidermal cells are thinner than wool fiber epidermal cells. The structure has irregular forms, such as a cube, an inclined ring or tile, and a crack-like shape. During the stretching process, it can easily be broken.

The strength of the alpaca fibers is close to sheep wool. The elongation properties of these fibers are 10% more than that of sheep fibers. The cross-section area of these fibers is not round, and channels consist of a core type of structure at the center of the fibers, making alpaca lightweight as compared to wool fiber. Sustainability, ecological design, and eco-efficiency are driving the development of the next generation of materials. Therefore, this trend is encouraging the manufacturing of innovative, high-performance materials at affordable prices to meet society’s basic needs. Thus, researchers and businesses are focusing their efforts on the creation of biodegradable and sustainable products made from natural fibers, which now have exceptional degradable and renewable properties. These products are biocompatible and biodegradable, and they are made from sustainable biomass feedstock, which can replace conventional polymers and reduce the world’s dependence on fossil fuels in certain applications [28,29]. Furthermore, polysaccharides, such as cellulose and keratin (wool, hair, and chicken feather), are abundant on Earth [29]. Keratins (KER) are a group of cysteine-rich fibrous proteins found in materials like wool, hair, chicken feathers, and nails [30]. Wound treatment, tissue regeneration, cell seeding and diffusion, and drug delivery are all considered to benefit from these materials as topical or implanted biomaterial devices [31]. KER has been the focus of extensive research due to the regeneration aspect of wool, hair, and feathers, as well as their ability to easily be converted into biomaterials [32]. Because of their widespread accessibility, light weight, low cost, environmentally-friendly nature, and insolubility in organic solvents, these materials also show some good characteristics for hydrophobic behavior and the ability to dampen sound [33]. Fibers obtained from chicken feathers, also known as CFF, for example, offer a broad, low-cost market as an additive for medium-density fiber board (MDF). Half of the feathers are fiber and half are quills (by weight); both the fiber and the quill are made of hydrophobic keratin, a protein with a tenacity equal to nylon and a diameter smaller than wood fiber [34]. The fiber is thicker than the quill and has a higher volume fraction. Human hair, like other natural keratin, which is made up of proteins and long chains (polymers) of amino acids, is the main component of hair fiber [35,36]. These days, human hair composites are widely used in the areas of construction, automobiles, and in reshaping furniture [37,38,39]. One of the other most important animal fibers with good thermal properties is wool fibers, which differ greatly from cotton and other fibers. There is less research available on composites made from animal fibers, but there is a lot of research on natural fibers obtained from plants. In this article, various animal fibers and their applications are discussed.

## 2. Different Categories of Animal Fibers

Animal fibers, such as silk, wool, fur, and feathers, are the second most important natural fiber source for composite reinforcement after plant fibers due to their easy availability and non-toxic nature. Moreover, animal fibers make biodegradable and eco-friendly combinations that provide a path to eliminate major solid waste materials. Wool comes from sheep, alpaca, bison, cashmere, muskox, and other animals, and each form of animal fiber has many sources. Silk, fur, and feathers are all common choices, according to several sources. Researchers examined at the use of animal fibers as reinforcement in composites and found that wool is commonly used in the textile industry for a variety of purposes, while chicken feathers are a waste product collected from slaughterhouses and could be used as reinforcement with various polymers as a matrix in fabrication of various composites [40,41].

### 2.1. Chicken Fibers

The waste from the poultry industry, mostly the chicken, constitutes four billion pounds annually in United States alone. A total of 20 billion chickens are killed around the world every year, constituting 4 billion pounds of poultry feathers [42]. This large number of waste feathers not only pollute the soil and air, but is also responsible for many human ailments, such as chlorosis, mycoplasmosis, and fowl cholera. As a result, studies on the use of CFF as main constituents in composite design have been ongoing for the past two decades, and several attempts are currently being made to turn this waste into useful items, as these feathers have some unique properties, such as low relative density and strong thermal and acoustic insulating properties. Furthermore, these fibers are inexpensive, and the amino acid of a CFF is nearly identical to that of other feathers. These fibers are durable, solid, lightweight, and have good thermal and acoustic insulating properties because the amino acids in them are cross-connected with each other by forming disulfide or hydrogen bonds [43]. The composition of these fibers constitutes around 91% protein (keratin), 1% lipids, and 8% water [44]. Feathers have a highly ordered structure, with a hierarchical branched structure that ranks among the most complex keratin structures found in vertebrates. Contour feathers are important because they provide the birds with a variety of colors, and they also serve as a protection against physical objects, sunlight, wind, and rain. These feathers are found on the bird’s backside [45]. Winandy et al. [46] studied the effect of feather fiber–wood fiber mixtures on composite panels; the fiberboards were fabricated by adding chicken feather fibers and formaldehyde resins were used as an adhesive. The mechanical properties of fabricated composite were evaluated, and it was observed that there was some loss in strength and stiffness of the new material when compared to the all-wood panels, but there was significant improvement in the resistance to water absorption and thickness, which is due to the hydrophobic keratin in the feather fiber. The mechanical properties of feather barbs were studied statistically by Zhan et al. [47], and the mechanical properties of CFFs were found to be beneficial for their various applications in composite materials, with significant differences in tensile modulus and tensile strength among different fibers.

**Figure 1:**
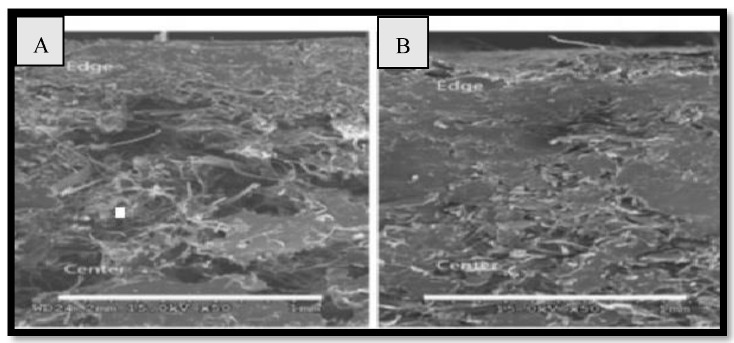
SEM images of the cross-sections of ground quill–PP composites at a temperature of 195 °C with a ground quill concentration of (**A**) 20% and (**B**) 35%. Reprinted/adapted with permission from Ref. [48].

**Figure 2:**
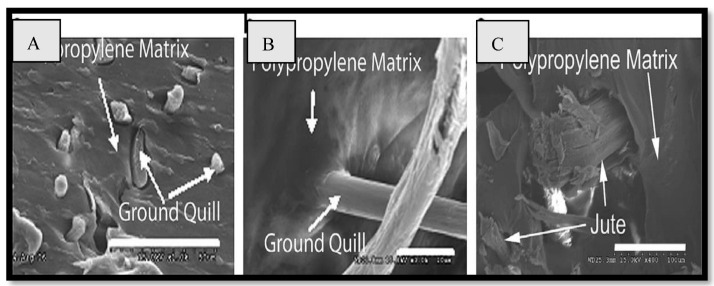
SEM images of (**A**) ground quill–PP composite, (**B**) ground quill–PP composite at 3.0 K·magnification, and (**C**) jute–PP composites. Reprinted/adapted with permission from Ref. [48].

**Figure 3:**
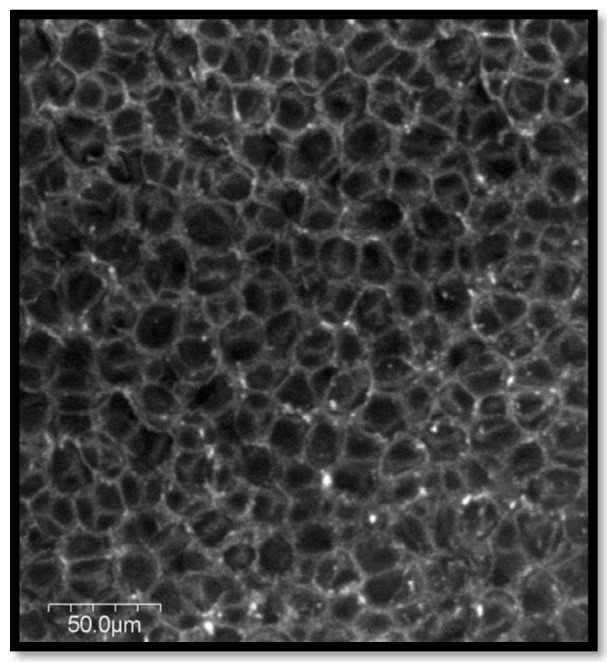
SEM image of ultra-thin quill cross-section showing honeycomb-shaped air pockets. Reprinted/adapted with permission from Ref. [48].

The mechanical and acoustic properties of chicken quill and polypropylene (PP) composites, as well as the effect of soil quill concentration, sustaining temperature, and density on structural mechanical properties, and the impact of soil quill concentration and density on sound absorption were investigated by Huda et al. [48]. The inner voids in the quill are shown in Figure 1 and Figure 2. The results revealed that quill composites have a much higher noise reduction coefficient (NRC) than jute composites. Figure 3 shows the ultra-thin quill cross-section with honeycomb-shaped air pockets.

**Figure 4:**
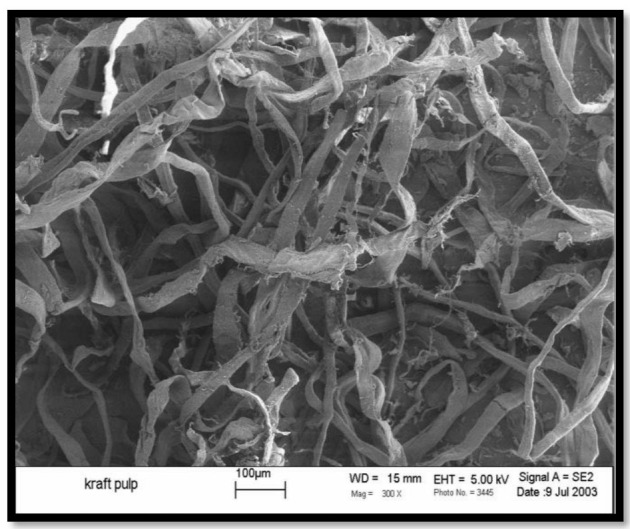
SEM image of kenaf bast fiber. Reprinted/adapted with permission from Ref. [49].

**Figure 5:**
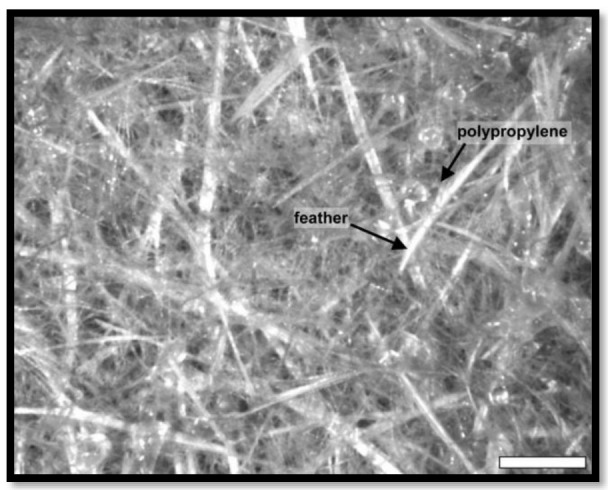
Images of wet lay prepreg after heating in a convection oven. Reprinted/adapted with permission from Ref. [49].

Bullions et al. [49] manufactured composites consisting of different compositions of feather fiber (Ff), recycled kraft pulp fiber (Pf), recycled newspaper pulp fiber (Nf), and retarded kenaf bast fiber (Kf) using polypropylene as a matrix. Compression molding was used to produce the fibers. Tensile and three-point bend tests were carried out on the new composites to determine the mechanical properties of the material. The contributions to the composite intensity of the four separate fibers were found to be, from the highest to the lowest: Pf > Nf > Ff > Cf. Figure 4 shows the kenaf bast fiber and Figure 5 shows the wet lay prepreg after heating in a convection oven.

Amieva et al. [30] fabricated recycled polypropylene composites reinforced with quill from chicken feathers with a 5, 10, and 15 percent wt reinforcement. The quill displayed a good distribution and impregnation into the PP matrix due to its hydrophobic nature. Quill material greater than 5% wt causes a matrix overload, which decreases the appropriate load transfer, as seen in the DMA analysis, but improves the dynamic storage modulus of the composites in all cases. The analysis indicates that all the tests demonstrate that small quill quantities (5–10 percent wt) are adequate to properly reinforce polymer matrices [50]. In another study, Cheng et al. [51] explored the varied mechanical properties of CFF with PLA. The fibers were manufactured using the process of injection molding. It was observed that CFF/PLA composite tensile modules were significantly higher than pure PLA. The results of DMA showed that the composite storage modulus enhanced with respect to the pure polymer, while the mechanical loss factor (tan d) decreased. In addition, the findings of the TGA experiments showed that the addition of CFF improved the composites’ thermal stability relative to pure PLA. The influence of a chemical treatment on the physicochemical and tensile properties of turkey feather was investigated by Oladele et al. [52]. A treatment of CFF was carried out using H_2_O_2_, NaOH, and KOH, and it was observed that the physicochemical and tensile properties of the treated fibers were significantly improved after the surface treatment.

**Figure 6:**
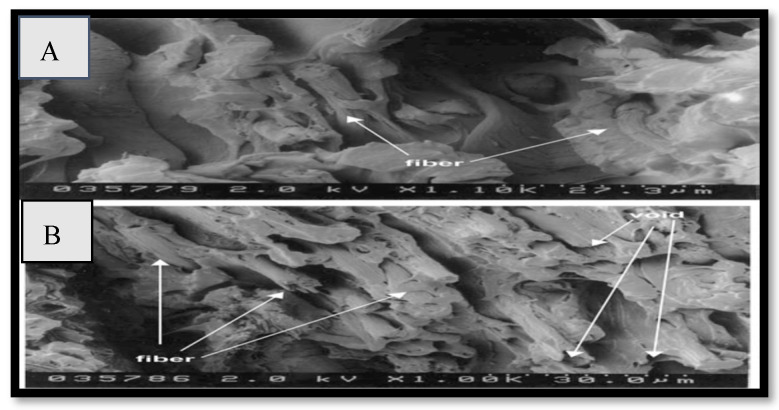
(**A**,**B**) Keratin feather fiber in LD133A LDPE. Reprinted/adapted with permission from Ref. [53].

**Figure 7:**
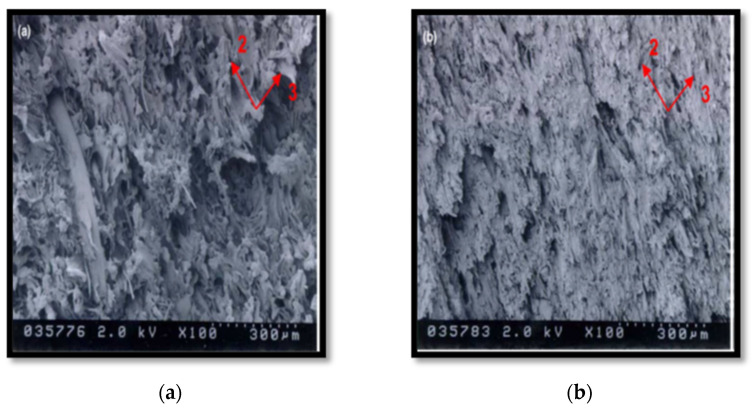
(**a**,**b**) SEM images of keratin Fiber. Reprinted/adapted with permission from Ref. [53].

Barone et al. [53] used CFF and polyethylene to prepare composites. Fibers of equal diameter but variable aspect ratio were mixed into low-density polyethylene using a mixing head (LDPE). As shown in Figure 6, a significant improvement was observed in an elastic framework and the yield stress of the newly produced fiber. The SEM results show that the polymer and keratin feather fiber interacted well, as shown in Figure 7. It can be concluded that CFF has a large possibility for advancement and discovery through innovative research in the field of advanced composite materials. Along with CFF as a matrix, particulate, or fiber, other hybrid bio-composites can be fabricated for sustainability. Moreover, highly improved hybrid materials can be designed by modifying existing materials and enhancing their properties. Furthermore, if the process of mixing fiber with matrix during casting is improved, an improved characterization of the generated composite with much more cost-effective applications can be achieved using CFF.

### 2.2. Silk

Silk fibroin in nature has evolved to adapt to their complicated habitats, such as humid or submerged settings, and to attach to a variety of surfaces. Bombyx mori, or silk fibroin, has long been used in a variety of applications. There are a variety of silk fibroin compositions found in nature. Silkworms develop protein fibers whose structure is dependent on two proteins: fibroin and sericin [54]. Spider silk is a natural polymeric fiber with high tensile strength and toughness, and has distinct thermal, optical, and biocompatible properties. Its primary structure consists of amino acids with characteristic sequences of glycine, alanine, serine, valine, and tyrosine and its strength and resilience is due only to the presence of these elements [55,56]. Many insects and most butterfly larvae (approx. 140,000 Lepidoptera) generate silk during metamorphosis [57]. Besides that, over 40,000 known spider species use up to seven different types of silk for shelter, defense, prey capture, and reproduction, as well as a dragline during their entire lifespan. Table 2 describes several species producing selected silk.

**Figure 8:**
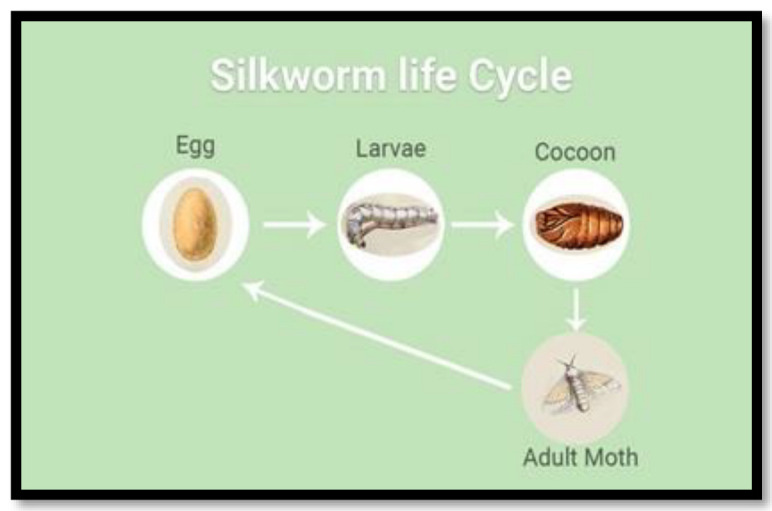
The different stages of silkworms.

Natural silk has many varieties that are well-known and commercially produced. Mulberry silk is one of the most popular, accounting for up to 95% of global production. The silkworm, such as a typical butterfly (Lepidoptera) insect, goes through four stages in its life cycle: egg, larva, pupa, and adult, as depicted in Figure 8. The duration will last anywhere from 6 to 8 weeks, depending on the weather. Table 3 highlights the tensile properties of the selected synthetic and natural fibers.

For fiber processing and textile applications, the sericin proteins, also known as silk gum, must be washed away. They serve as an adhesive connecting the layers of continuous silk fiber. Mulberry silk fibers are semi-crystalline polymers made up of sheet crystallites contained in a protein matrix [62]. The tensile strength of mulberry silk is 0.6 GPa, which is higher than that of other plant fibers [63]. Mulberry silk is resistant to mild acids and is insoluble in most alcohols or acetone (CH3COCH3, propan-2-one). It has small water swelling and negligible water absorption. Hydrochloric acid, on the other hand, necessitates a few hours of hydrolysis exposure, which happens primarily in the amorphous regions.

**Figure 9:**
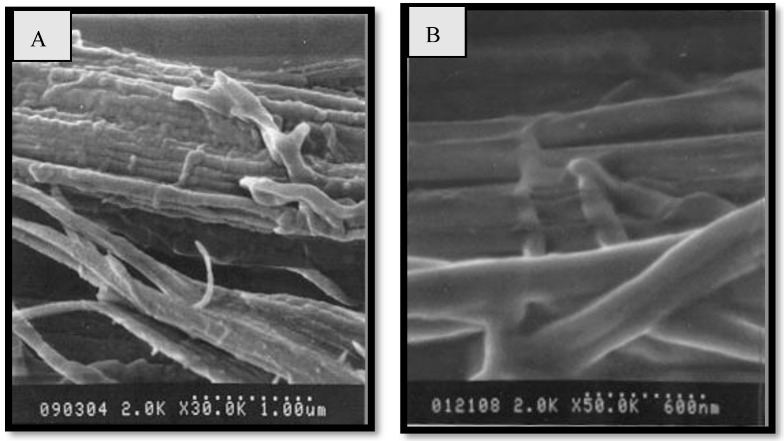
SEM images of peeled silks; (**A**) *A. pernyi* and (**B**) *A. yamamai*. Reprinted/adapted with permission from Ref. [64].

The heavy chain consists of twelve repetitive domains forming the fiber’s crystalline regions interspersed with less structured domains forming the amorphous parts [64]. The other essential silk is dragline silk (Nephila), a biomaterial with amazing mechanical properties, resulting in numerous potential applications and developed by a spider during its lifetime. It has long amazed scientists with its high strength and ability to stretch before breaking. Spiders use up to seven different forms of silk for shelter, defense, prey capture and reproduction as compared with other insects during their lifetime [65]. Each of these varieties of silk is highly adapted to their function and developed in a specialized gland. It is the strongest and hardest fiber in the silk package of the nephila spider with a diameter of about 3–5 μm [66]. Like mulberry silk, dragline silk is a semi-crystalline polymer composed of β-sheet crystallites embedded in an amorphous protein matrix. The primary structure of the silk polymer is, to some extent, predefined by both amorphous and crystalline parts. The mechanical properties of dragline silks over a large temperature range are almost constant and give an amazing response to low temperatures, whereas they marginally decrease at temperatures rising from the freezing point to almost 150 °C, which is where degradation begins. This type of behavior is comparable to polyamide 6.6 and mulberry silk [67]. However, with temperatures falling below the freezing level, the mechanical properties greatly improve. At −60 C average, the extensibility reaches almost 45%, while the tensile strength exceeds that of steel at around 1.5 GPa [68]. SEM images of peeled silks are depicted in Figure 9. Table 4 highlights the properties of mulberry and nephila dragline silk and Table 5 shows the list of organisms successfully modified to produce spider silk protein.

In the presence of ACAC, Zhou et al. [75] enzymatically grafted copolymerized acrylic acid with silk fibroins and H_2_O_2_- HRP. The particle size of the SF–g–PAA copolymer was slightly higher in comparison to the untreated fiber. The mechanical properties of the mineralized SF–g–PAA composite membrane were also improved as compared to the untreated. The mineralized SF–g–PAA and SF composite membranes have aided osteoblast cell adhesion and proliferation. The SF–g–PAA composite has the potential to be employed as a biomaterial for bone tissue engineering. Moreover, in another study, Wang et al. [76] studied the process of tyrosinase-catalyzed grafting It was observed that chitosan could be adsorbed by electrostatic interactions and other weak forces on silk fibers, while the amine groups of chitosan that could react with o-quinone residues oxidized from the tyrosyl residues of silk fibers, which can improve the strength and crease-resistant ability of silk fabrics.

In another study, Lu et al. [77] explored the mechanism of silk fibroin degradation based on films with different secondary formations and nanostructures. It was found that the hydrophilic blocks were first degraded during the degradation cycle, allowing stable blocks with a high content of crystal structures to transfer to protease solutions and silk fibroin films with the lowest β-sheet content to achieve the highest degradation rate. Furthermore, Wang et al. [78] covalently grafted onto the surfaces of the fibroin using 1-ethyl-3-(3-dimethylaminopropyl) hydrochloride carbodiimide (EDC) to increase the reactivity of silk fibroins. Mushroom tyrosinase was used to oxidize the modified fibroins enzymatically. This was accompanied by a coupling of π-polylysine (ÿ-PL) with the silk fibroin o-quinone residues produced. The results suggest that treatment with EDC may also induce the direct self-crosslinks of silk fibroins and TyrP-bridged cross-links of fibroin molecules, leading to a significant increase in fibroin protein molecular weight [79].

**Figure 10:**
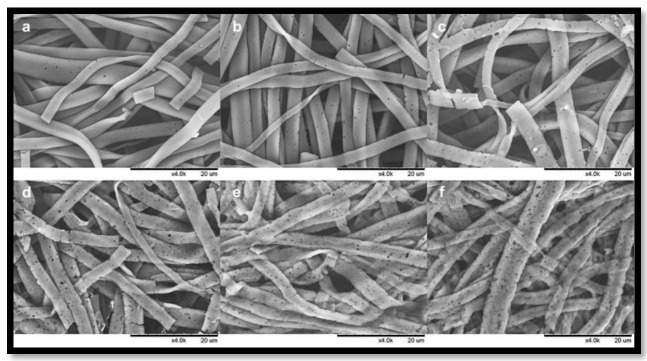
SEM of the SF scaffolds during degradation in 1.0 U/mL protease XIV. (**a**) 1 d, (**b**) 3 d, (**c**) 6 d, (**d**) 12 d, (**e**) 18 d, (**f**) 24 d. Scale bars ¼ 20 mm. Reprinted/adapted with permission from Ref. [79].

**Figure 11:**
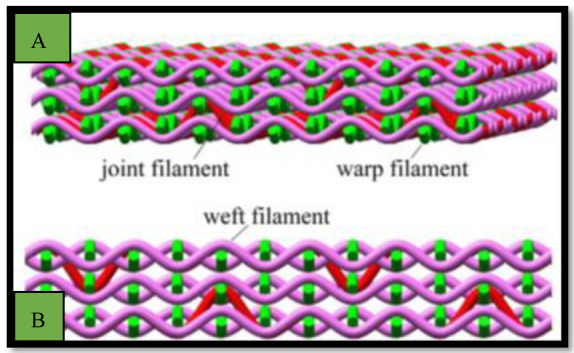
(**A**) 3D and (**B**) cross-section schematics of MLNFF scaffolds. Reprinted/adapted with permission from Ref. [80].

Shao et al. [80] fabricated a scaffold composed of multilayer nanofiber fabrics (MLNFFs). The 3D cross-section shown in Figure 10 and Figure 11 demonstrates that this scaffold was developed by weaving polylactic acid (PLA) nanofiber yarns and tussah silk fibroin (TSF) by an electrospinning process. The scaffolds were based on a 90:10 mixture of PLA and TSF electrospun into nanofiber yarns with a uniform distribution of diameter and strong tensile strength. In vitro studies suggest that not only does the woven scaffold promote MSC adhesion and proliferation, but it also increases ontogenesis, production of alkaline phosphatase, and mineralization. The scaffolds are therefore ideally suited to bone tissue engineering [81]. Therefore, from the section above, it can be concluded that attempts are being undertaken to regulate the silk fibroin-based technology from the ground up and to encourage its industrialization from the laboratory scale by creating biotechnology and sustainable production methods and protocols, as it is difficult to obtain a large quantity of natural spider silk because spiders are aggressive and tend to kill each other if they are raised in a limited space. Therefore, artificially mimicking the internal structure of natural spider silk is essential. Moreover, increasing a fiber’s strength and toughness is a long-term goal in the field of fiber materials. Researchers have investigated ways to increase the mechanical qualities of spider silk.

### 2.3. Wool

The complex molecular and morphological structure of keratins is similar to the building concept of biological composite structures, which is to mix components with varied qualities in one material to optimize compatibility for its function [82]. Single wool fibers are extremely heterogeneous in composition and shape; they are, essentially, a biomaterial dependent on a multicomponent. Wool fiber is made up of three layers. The outermost layer of scales is called the cuticle, the middle layer is called the cortex, and the inner center is called the medulla [83]. This protein is insoluble in water, organic solvents, diluted acids, and alkalis, and in common solvents, it shows resistance to degradation. This is because of the tight packing of the α-helices and β-sheets present in the wool keratin’s polypeptide structure [84]. The diameter of the fibers varies significantly among different sheep breeds (between 11.5 and 47 μm) [85,86]. Table 6 shows the amino acid composition of wool, cashmere, and yak fibers.

The complex morphological and molecular structure of α-keratins echoes the construction principle of biological composite structures in general, namely, to combine components with different properties in one material to maximize its suitability for its purpose. Of interest is the recovery of the fiber after a strain of up to 30%. There are several models proposed for describing the stress–strain curve of wool and the hysteresis behavior, with Fogelman’s model being the most well-known [88]. In both cases, the mechanical effort is seen as being distributed between the α-helix of the crystalline region of the fiber (IFs) and the amorphous matrix. Their contributions depend strongly on the moisture content of the fiber, with the matrix effect almost vanishing at 100% relative humidity. Table 7 shows the mechanical properties of wool fiber at 22 °C.

The paracrystalline filaments will resist shrinkage after drying so that the length change at 1–2 percent is small when rewetting or swelling from the dry state. Because of the protein structure, wool absorbs a significant amount of moisture that binds to the amino acids by means of hydrogen bonds, reaching 33 percent of its dry mass, the highest percentage among natural fibers. Since the crystalline region is water-impermeable, the amorphous matrix can absorb water up to 45 percent of its dry mass (for a 25 percent crystallinity) without feeling moist [81,87]. The sheep is the most important of all producers of keratin fibers. Wool fibers are typical raw materials for textiles; they are classified for the clothing industry or for the interior textiles industry according to the fiber diameter. Table 8 shows the composition in % of greasy wool.

Zach et al. [89] studied the appropriateness of wool in buildings as thermal insulation and tested the wool under different conditions. The findings showed that sheep wool thermal insulation has comparable characteristics with mineral/rock insulation and performs even better in some applications. In another study, Jeon et al. [90] developed a method of altering hydrophobic thread wettability for wool thread. Plasma treatment for thread-based microfluidic devices was applied to wool threads. DBD plasma, with different gases, viz. oxygen, argon, nitrogen, and air, was evaluated on wool fiber. The analysis indicates that there was an improvement in wettability due to the removal of the fatty acid layer on the outermost cuticula surface. SEM images were obtained to investigate the morphology of surfaces. Cui et al. [31] explored the impact of TGase, an enzyme that catalysis acyl transfer processes by forming covalent cross-links between proteins to increase protein stability, on the characteristics of keratin films. Cell growth and drug release was tested on the TGase-modified film. The corrected sample’s tensile strength rose, according to the findings. The film’s stability in PBS and artificial gastric juice was improved as well. Furthermore, Goudarzi et al. [91] studied pronase, trypsin, papain, and pepsin enzymes for use in the textile industry and treated the wool using these enzymes at optimum conditions for 30, 60, and 120 min. It was concluded from the results that papain is more proteolytically efficient for wool fiber morphology.

### 2.4. Human Hair

**Figure 12:**
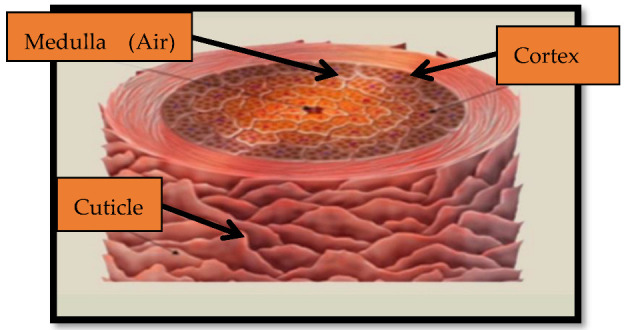
Parts of hair fiber [37].

Human hair is a non-biodegradable waste that is commonly available around the world, yet it is rarely considered for engineering purposes. Human hair surface tensile strength varies from 150 to 220 MPa. Human hair is a fibrous substance that has excellent tensile properties. The hair fiber’s primary component is keratin, which consists of proteins and long amino acid chains (polymers). Figure 12 shows the components of human hair fiber [92]. Keratin is the main constituent of fiber in human hair. These keratins are proteins and amino acid polymers. The cytoskeleton of all epidermal cells is generated by keratin proteins. Keratin proteins make up 65–95 percent by weight of the total hair fiber. Rao et al. [37] produced bio-composites by manually laying down local hair and polyester and investigated the effect of the fiber volume fraction on the physical parameters (density, porosity) and tensile strength of the randomly oriented composite. Composites with a low fiber volume fraction are prone to intra-fiber voids, while composites with a high fiber volume fraction are prone to inter-fiber voids and have a maximum tensile strength of 23.5. Hair is stunningly strong and cortex keratin is responsible for this property, and its long chains are compressed to form a regular structure that is flexible, in addition to being solid. This property is used extensively to make this composite material more flexible [93]. The keratin-protein peptide chain is arranged as a right-hand α-helix, which coils further to form proto fibrils [94]. Hair strength is determined by the protein structure, ageing, and chemical impacts, as well as its vulnerability to mechanical (combing and curling) and heat stresses (drying and straightening) [81]. Although the cortex determines the tensile behavior of human hair, we now know that the non-keratin elements of the cuticle and the cell membrane complex also affect the fiber’s physical integrity to combing and grooming stresses [95]. Table 9 highlights the amino acids present in normal human hair and Table 10 shows the amino acids from frosted vs. non-frosted hair.

Bongu et al. [98] suggested a new formulation of nanocomposites in which Mn_2_O_3_ nanoparticles were wrapped in N and S containing randomly focused carbon-like graphene sheets (HHC). These can be generated using a quick, scalable, and easy method using human hair. The Mn_2_O_3_/HHC nanocomposites were prepared and found to be superior to any other Mn_2_O_3_ anode material previously reported. The analysis indicated that the Mn_2_O_3_/HHC anode exhibited a reversible lithium storage capacity of 990 mAh g^−1^ over 350 cycles at 50 mA g^−1^ and 440 mAhg^−1^ over 2000 mA g^−1^ (2C), which is the world’s highest value for a Mn_2_O_3_ anode in the literature.

In another study, Deepmala et al. [99] fabricated human hair reinforced with soy protein isolate (SPI)-based green composites and changed those composites with 40 wt. phytagel and 12.5 wt. glycerol percent. The surface morphology was investigated using SEM. Tensile tests showed that the composites with a maximum tensile strength of 2 wt. percent of human hair fiber 17.23 MPa were obtained, while neat SPI tensile strength was 8.54 MPa. In a similar study, Senthilnathan et al. [93] manufactured the human hair hybrid glass fiber–coconut fiber composite using epoxy resin as a reinforcing agent. This composite was developed using a manual layout method. The laminates were prepared using LY556 resin and HY951 hardener, and the various mechanical properties, such as tensile, flexural, shear, impact, and hardness testing were evaluated. The findings showed that the mechanical properties were enhanced along with human hair in the glass fiber laminates.

## 3. Applications of Animal Fibers

Renewable animal fibers provide an attractive prospect for the development of sustainable bio-composite materials. Researchers’ focus on these animals has recently risen due to their ease of availability, light weight, low cost, and sustainable nature. Chicken feathers, human hair, and hair from other birds and animals are all generally waste by-products, but these fibers can play integral role in the future to decrease the dependency on synthetic fibers. The fibers obtained from animals in the form of wool and silk are important for use in various sustainable applications. Furthermore, owing of the existence of hydrogen bonding and the hydrophobic structure of the protein, these fibers are more stable than spherical proteins. Silkworms provide silk fibers to regenerate tissues for biomedical applications. Moreover, these fibers can be used in the automobile industry, as these renewable fibers can be used as reinforcements in composites of the interior parts for a number of passenger and commercial vehicles [100,101,102]. In particular, chicken feathers offer the specific advantage of low relative density and strong thermal and sound insulating properties. On an other hand, human hair has sufficiently high strength for use as sutures in most surgeries. Studies have shown the potential of human hair sutures in cataract and conjunctival wound repair surgeries and general surgeries on humans and animals [103]. In the next section, various applications of animals fibers are discussed.

### 3.1. Biomedical Applications

Today, materials science is undergoing a paradigm change in the production of modern smart devices for biomedical uses. Indeed, there is a clear need for novel approaches capable of addressing and overcoming degenerative and highly misrepresented diseases affecting a rapidly increasing number of people, often due to the population’s rapid ageing. Chicken feathers regenerate hard tissue and offer the specific advantage of low relative density and strong thermal and sound insulating properties. They can be used in a variety of applications, as billions of chickens are harvested each year. Moreover, technologies are established and patented for biomedical applications for the processing of chicken feathers into fibrous (feather fiber) and particulate (quill) fractions.

Reddy et al. [103] studued chicken fibers for use in tissue engineering using the compression moulding process. Fibers are biocompatible and have cross-linking characteristics because the main protein is biocompatible. The thermoplastic films from the features were created and analysed, with the results revealing that the feather films were water resistant and strong [104,105,106,107,108,109]. This could be utilised to make biomaterials for a variety of biomedical applications [110,111,112,113]. Human hair has sufficiently high strength for use as sutures in most surgeries. Studies have shown the potential of human hair sutures in cataract and conjunctival wound repair surgeries and general surgeries on humans and animals, as well as in microsurgery [113,114,115].

### 3.2. Constructional

Reddy et al. [116] examined various applications of silk fibers for the construction industry and observed that silk fibers have economical and technical advantages of having insulative properties higher than the current materials used. Moreover, the mechanical properties of silk fiber and polypropylene (S–PP) composites were found superior to standard glass fiber reinforced plastics (GFRP). Dweib et al. (2004) manufactured unit beams using chicken feathers. The manufacturing was carried out using vacuum-assisted resin transfer molding (VARTM) technology. It was observed that the natural fiber reinforcement of 20–55 wt. % fiber enchanced the mechanical properties of the structure [117].

Gupta et al. [38] examined the used of human hair in plastering house walls, lining ovens, making wheels, and so forth in parts of the world, as it considerably minimizes cracking and extends the life of these structures. Savio et al. [118] fabricated hemp and wool insulation mats for sustainable buildings as semi-rigid products with an environmental impact. The panels are ideal for the eco-building sector since they are 100 percent recyclable and created from by-products from local manufacturing chains (Piemonte Region). Acada et al. [119] studied the environmental thermal insulation properties of sheep wool and observed that it can be used as insulation for buildings in the thermal conductivity absorption of formaldehyde.

### 3.3. Automobile Applications

The use of bio-based materials in automotive parts was initially considered by Ford Motor Company founder Henry Ford in the 1930s [120]. In 1942, Henry Ford began using natural fibers in the automotive sector by experimenting with soybeans to form fine plastic components. According to experts in materials from various car manufacturers, an all-advanced composite auto body will be 50–67% lighter than an existing, similarly sized steel auto body, compared to a 40–55% reduction in mass for an aluminum auto body and a 25–30% reduction in mass for an engineered steel auto body. Furthermore, using keratin fiber and plant fiber reinforced composites has several advantages, including a high diameter-to-length ratio and flexibility [121,122].

Wool and silk fabric for automobiles is a significant part of the textile industry. The use of natural fibers in the manufacture of textile materials for automotive interiors is limited to wool [123]. Huda et al. [124] examined the various properties of chicken feathers for applications in automobile air filters, and the results of various experiments revealed that satisfactory results can be achieved after treating chicken feather fibers individually with two or three sets of high-consistency disk refiner in the series, and the super fiber characteristics of feather fibers makes the fibers appropriate for manufacturing automobile air filter paper.

## 4. Conclusions

The ability of green composites to serve as alternatives to synthetic fiber-reinforced polymer composites has attracted significant attention due to their availability, non-toxic nature, and non-corrosive properties. Substantial research on these fibers and their composites is currently ongoing globally to develop the necessary properties [125,126]. Fibers and composites are classified according to their characteristics and uses in various applications. Sustainable animal fibers offer an attractive prospect for the development of sustainable bio-composite materials. Because of their simple availability, light weight, low cost, and ecologically favourable design, researchers are now focusing more on these animal fiber-reinforced composites. There has been little research performed on composites made from animal fibers, but there is a lot of research available on natural fibers made from plants. Human hair, chicken feathers, and other bird and mammal hair are usually classified as waste by-products, which can be utilized for sustainable applications to impede climate change that poses a huge threat to our environment. Moreover, the waste will be also used in a productive way. Keratin is a form of protein that is high in sulfur-containing amino acids like cystine. It is the major structural fibrous protein that gives mammals, reptiles, and birds their exterior coverings, such as hair, wool, and feathers. Further research into the processing of regenerated materials into fibers and films, as well as the recycling of ILs, is now underway.

Future road map:

- A policy for extending the use of human hair while maintaining social and environmental norms can be framed by various governments.
- The enactment of rules and regulations, as well as the development of support structures for various uses of animal fibers based on their environmental impact, corporate requirements, and market reach can be framed.
- It is necessary to educate the community about the beneficial characteristics of animal fibers, as well as safe collecting and usage techniques.
- Complete human hair utilization systems may be established with the help of many stakeholders, reducing solid waste and environmental concerns, generating major social economic advantages for humans, and reducing pressure on other non-renewable resources and fossil fuels that can be saved for future needs.

## Figures and Tables

**Table 1:** Demonstration of the different properties of natural fibers derived from plants and animals [5,6,7,8,9,10,11,12,13,14,15,16].

Fibers	Properties	Diameter (µm)	UTS (MPa)	Elongation at Break (in µm)	E (GPa)	Density (g/cm^3^)
**Flax**	Lightweight, absorbent	12–16	300–1500	1.3–10	24.80	1.4–1.5
**Jute**	Strength, durability	17–20	200–800	1.16–8	10–55	1.3–1.5
**Sisal**	Strength, durability	200–400	80–840	2–25	9–38	1.5
**Kenaf**	Rough	25–35	296–1191	3.5	2.86	-
**Abaca**	Thin, lightweight	40	980	-	7.31 × 10^−4^	-
**Pineapple**	Soft, lightweight	10–28	170–1627	2.4	60–82	0.8–1.6
**Banana**	Warm, thick, durable	200	529–914	3	27–32	-
**Coir**	Strength, durability	10–20	106–175	14.21–49	4–6	1.2
**Ramie**	Heavy, tough	20	348–938	1.2–8	44–128	-
**Hemp**	Strength, durability	16–50	310–900	1.6–6	30–70	1.48
**Wool**	Warmth	16–40	120–174	25–35	2.3–3.4	-
**Spider silk**	Smooth fabric finish with high shine	10–13	875–972	17–18	11–13	-
**Cotton**	Lightweight, absorbent	11–22	264–800	3–8	5–12.6	-
**Mulberry silkworm fiber**	White-toned and more reproducible	10	208.45	19.55	6.10	1.33
**Wild (Tussah) silkworm fiber**	Beige to brownish-toned	25	165.27	20.57	3.82	1.32
**Twisted B. mori silk**		10	248.77	33.48	5.79	-
**Camel hair**	Softness, warmth	20.04	212.15	37.05	3.87	-
**Catgut fiber**		790	100	-		
**Angora wool**	Softness, thin fibers	12–16				
**Yak fiber**	Warmth, softness, breathability, odor-resistant	15–19	270.05	14.53	45.0943	3.41
**Alpaca**	Lightweight, soft, fine, glossy, and luxurious	12–29	53.5	42.3		1.38
**Bison**	Red brown, soft	59				
**Llama**	Fine, soft	30–40				
**Qiviut**	Long, smooth, 8 times warmer than sheep	15–20				

**Table 2:** Silk-producing species, the name of their silk, and their diet and domestication grade [10,58,59,60,61].

Species	Silk	Diet	Domestication
Bombyx mori (silkworm)	Mulberry (cocoon)	Morus spp. (mulberry) exclusively	Yes
Antheraea pernyi (Chinese oak silk moth)	Tussah (cocoon)	Quercus spp. (oak) exclusively	Semi
Antheraea assamensis	Muga (cocoon)	Machilus bombycina Latsaea polyantha	Semi
Philosamia cynthia ricini	Eri (cocoon)	Ricinus communis (castor) exclusively	Yes
Nephila sp. (Golden orbweaver)	Dragline (among others)	Insects	No
Araneus sp. (European garden spider)	Dragline (among others)	Insects	No
Trichoptera sp. (Caddiesfly)	Part of cases	Herbivorous Carnivorous	No

**Table 3:** Tensile properties of selected man-made and natural fibers [62].

Material	Tensile Strength in GPa	Extensibility (% of Initial Length)	Young’s Modulus in Gpa	Toughness in MJ/m^3^
High-tensile strength steel	1.5	0.8	200	6
Aramide (Kevlar)	47	3.6	2.7	130
Polyamide 6.6 (Nylon, DuPont)	0.95	18	4	80
Mulberry silk (Bombyx mori)	0.6	18	6	70
Dragline (Nephila)	1.1	30	20	170

**Table 4:** Properties of mulberry and Nephila dragline silk [66,69,70,71,72,73,74].

Property	Bombyx Mori	Nephila Dragline
Degree of crystallinity in %	38–66	20–45
Density in g/cm^3^	1.35–1.42	
Crystallite size in nm	1.0–2.5	4.7 × 5.3 × 6.0
Index of refraction	1.591 parallel to fiber	1.538 perpendicular to fiber
Maximum use temperature in °C	170	150
Thermal degradation in °C	250	234
Heat capacity in J/g K	1.38	
Glass transition temperature	178 °C at 0% RH	39 °C at 75% RH
Super contraction in water	No	∼50%

**Table 5:** List of organisms successfully modified to produce spider silk protein [75].

Species	Year-Round Production	Advantage	Disadvantage
Capra aegagrus hircus (domestic goat)	Yes	Easy to keep and can be constrained in stables	High space consumption
Escherichia coli	Yes	Can be kept constrained in high densities	Only 30 kDa proteins
Nicotiana tabacum (tobacco)	No, seasonal	100 kDa proteins	Poor acceptance in Europe (genetic engineering for agriculture)
Solanum tuberosum (potato)	No, seasonal	100 kDa proteins	Poor acceptance in Europe (genetic engineering for agriculture)
Bombyx mori (silkworm)	No, seasonal	Produces fibers and raw protein, easy to keep and to keep constrained	Fibers are not pure spider silk protein, exclusive diet of mulberry leaves = mulberry plant age required

**Table 6:** Amino acid composition of wool, cashmere, and yak fibers [81,87].

Amino Acid in mol %	Wool	Cashmere	Yak
Glycine	8.1	9.9	9.8
Alanine	5	5.8	5.6
Serine	10.2	12.2	10
Glutamine + glutamic acid	12.1	12.4	12.5
Cystine	11.2	6	6.4
Proline	7.5	6.7	6.6
Arginine	7.2	7	7.1
Leucine	6.9	7.5	8.3
Threonine	6.5	6.6	6.6
Asparagine + aspartic acid	6	6.2	6.7
Valine	5.1	5.5	5.9
Tyrosine	4.2	3.5	3.4
Isoleucine	2.8	3.2	3.5
Phenylalanine	2.5	2.8	3
Lysine	2.3	2.8	3
Tryptophan	1.2	-	-
Histidine	0.7	1.2	1
Methionine	0.5	0.5	0.5

**Table 7:** Mechanical properties of wool fiber at 22 °C [89].

Breaking Stress	
Dry	250–350 MPa
Wet	100–200 MPa
Strength loss when wet	20%
Breaking strain	
Dry	28–48%
Wet	40–61%
Elasticity modulus	
Dry	4.0–5.0 GPa
Wet	2.0–3.0 GPa
Recovery at strain	
2%	95–99%
5%	60–70%
10%	40–50%
Bending modulus	4.0–5.5 GPa
Stretching modulus	5.0–6.0 GPa
Torsion modulus parallel	1.1–1.3 GPa
Stretching modulus/torsion modulus	3.0–4.0 GPa
Shear modulus in torsion	
Dry	1.2 GPa
Wet	0.1GPa

Note: ‘dry’ refers to 65% relative humidity (RH); ‘wet’ refers to 100% RH.

**Table 8:** Composition in % of greasy wool. The micron limits for the three wool types are only informative [88].

Wool Type	Grease and Suint	Sand and Dirt	Vegetable Matter	Fiber
Merino (<25 μm)	15–30	5–40	0.5–10	30–60
Cross-bred (25–33 μm)	15–30	5–20	1–5	40–65
Long wool (>33 μm)	5–15	5–10	0–2	60–75

**Table 9:** Amino acids present in normal human hair [96].

Amino Acid	Amount in Residues Extracted
Cysteine	17.5
Serine	11.7
Glutamic acid	11.1
Threonine	6.9
Glycine	6.5
Valine	5.9
Arginine	5.6
Aspartic acid	5
Alanine	4.8
Proline	3.6
Isoleucine	2.7
Tyrosine	1.9

**Table 10:** Amino acids from frosted vs. non-frosted hair [95,96,97].

Amino Acid	Micromoles per Gram of Hair		Significant Difference for
	Non-Frosted Fibers	Frosted Fibers	Frequencies at Alpha = 0.01 Level
Aspartic acid	437	432	_
Threonine	616	588	_
Serine	1085	973	_
Glutamic acid	1030	999	_
Proline	639	582	_
Glycine	450	415	_
Alanine	370	357	_
Half cystine	1509	731	Yes
Valine	487	464	_
Methionine	50	38	Yes
Isoleucine	227	220	_
Leucine	509	485	_
Tyrosine	183	146	Yes
Phenylalanine	139	129	_

## Data Availability

Not applicable.
